# The impact of acupuncture and moxibustion treatment in individuals with recurrent implantation failures: A systematic review and meta-analysis

**DOI:** 10.1097/MD.0000000000046587

**Published:** 2025-12-19

**Authors:** Junjun Chen, Yan Lyu, Xuzhen Cheng, Fang Zhang

**Affiliations:** a Reproductive Endocrinology Unit, Women’s Hospital School of Medicine Zhejiang University, Hangzhou, Zhejiang, China.

**Keywords:** acupuncture and moxibustion, meta-analysis, recurrent implantation failure, systematic review

## Abstract

**Background:**

The therapeutic efficacy of acupuncture and moxibustion in addressing recurrent implantation failure (RIF) remains a topic of debate. This systematic review and meta-analysis aims to synthesize current evidence on the benefits of acupuncture and moxibustion for patients with RIF.

**Methods:**

Embase, PubMed, Cochrane, Web of Science, CBM, WanFang, CNKI, and VIP databases were retrieved, with the search period spanning from each database’s inception to January 19, 2024, and an updated search was conducted on September 18, 2025. Studies were selected based on predefined criteria, and the quality was assessed using the RoB-2 tool. Data were analyzed using Review Manager5.3 software.

**Results:**

The meta-analysis included 15 studies, encompassing 1029 subjects. The results revealed several notable benefits of acupuncture and moxibustion in patients grappling with RIF. Statistically significant enhancements were observed in clinical pregnancy rates (risk ratio [RR] = 1.84, 95% confidence interval [CI] = 1.53–2.20, *P* < .05), live birth rates (RR = 2.39, 95% CI = 1.59–3.58, *P* < .05), endometrial thickness (mean difference [MD] = 1.37, 95% CI = 0.95–1.80, *P* < .05), endometrial morphology (RR = 1.67, 95% CI = 1.30–2.14, *P* < .05), and serum estradiol levels (standardized mean difference = 2.70, 95% CI = 0.20–5.21, *P* < .05) when acupuncture and moxibustion therapy was administered, compared to the control group. A subgroup analysis was conducted on the outcome of endometrial thickness, and the results showed that: interventions lasting for 3 menstrual cycles (MD = 1.62, 95% CI: 0.92–2.33, *P* < .001, *I*² = 92.4%), treatment using electroacupuncture combined with warm acupuncture (MD = 2.41, 95% CI: 2.05–2.77, *P* < .001, *I*² = 0%), and the use of staged acupuncture point selection (MD = 2.41, 95% CI: 2.05–2.77, *P* < .001, *I*² = 0%) were more effective in increasing endometrial thickness in patients with repeated implantation failure.

**Conclusion:**

In comparison to continuous oral estrogen and traditional hormone replacement therapies, acupuncture and moxibustion treatment has been found to provide more substantial advantages in enhancing clinical pregnancy rates, live birth rates, endometrial thickness, endometrial morphology, and serum estradiol levels among patients experiencing RIF.

## 1. Introduction

Infertility represents a significant global health issue, affecting around 10% to 15% of couples who struggle to conceive. As assisted reproductive technology continues to advance, in vitro fertilization and embryo transfer (IVF–ET) has become widely utilized for patients facing challenges in conception.^[1]^ Nevertheless, approximately 10% of patients undergoing IVF–ET experience repeated failures in transplantation worldwide, further exacerbating the economic and psychological burdens faced by infertile couples.^[2]^

Embryo implantation success hinges on the presence of a receptive endometrial lining, embryos that are developmentally appropriate at the blastocyst phase, and a well-timed exchange of signals between the mother’s and the embryo’s tissues.^[3]^ Nearly two-thirds of implantation failures in in vitro fertilization (IVF) are attributed to reduced endometrial receptivity.^[4,5]^ Current treatment options for improving endometrial receptivity in patients with recurrent implantation failure (RIF) include granulocyte colony-stimulating factor intrauterine infusion,^[6]^ platelet-rich plasma intrauterine infusion,^[7]^ and surgical improvement of the uterine environment. However, these approaches can be costly, and the efficacy of some pharmacological treatments is unsatisfactory and controversial.^[2]^ Acupuncture and moxibustion treatment is a prevalent method for treating female infertility, favored for its accessibility, effectiveness, and affordability.^[8,9]^ This traditional practice modulates multiple molecules associated with endometrial receptivity, impacting various pathways to improve microcirculation within the endometrium and thereby increasing its receptivity to embryo implantation.^[10,11]^ Currently, there are limited systematic evaluations assessing the effectiveness of acupuncture and moxibustion therapy in treating patients with RIF, and consensus on its intervention effects for individuals experiencing repeated transplantation failures has not yet been established.^[12]^ This article aims to update relevant literature in recent years through a systematic review and meta-analysis to explore the effectiveness of acupuncture and moxibustion therapy for RIF, thereby providing evidence-based references for its clinical use in the treatment of RIF.

## 2. Methods

This study adhered to the PRISMA guidelines for systematic reviews and meta-analyses,^[13]^ with its protocol dutifully registered within the International Prospective Register of Systematic Reviews under the identifier PROSPERO: CRD42024514409.

### 2.1. Search strategy

Computer searches were conducted in the Embase, PubMed, Cochrane, Web of Science, CBM, WanFang, CNKI, and VIP databases, with the search period spanning from each database’s inception to January 19, 2024, and an updated search was conducted on September 18, 2025. Chinese search terms included “recurrent implantation failure,” “acupuncture,” “RIF,” “randomized controlled,” “implantation rate,” “IVF outcomes,” and “assisted reproduction”; English search terms include “infertility,” “embryo transfer,” “recurrent implantation failure,” “RIF,” “acupuncture,” “randomized controlled trials,” “implantation rate,” “IVF outcomes,” “assisted reproduction,” etc. The search strategy incorporated a blend of subject-specific and unrestricted keywords, limited to studies published in Chinese and English languages.

### 2.2. Eligibility criteria

#### Inclusion criteria

The inclusion criteria were designed based on the Population, Intervention, Comparison, Outcome, Study Design (PICOS) elements from potential randomized controlled trials (RCTs).

(1). P (population): individuals who underwent at least 3 embryo transfers or had at least ten high-quality embryos transferred but failed to achieve a clinical pregnancy;^[14]^ or individuals diagnosed with RIF by medical practitioners or according to accepted diagnostic standards, such as individuals under 40 years of age who failed to achieve a clinical pregnancy after the transfer of a minimum of 4 high-quality embryos across 3 or more fresh or frozen IVF–ET cycles.^[15]^ All individuals were aged ≥ 18 years.
(2). I (Intervention): acupuncture and moxibustion treatment, including electroacupuncture, warming acupuncture, and standard acupuncture.
(3). C (Comparison): conventional Western treatments such as estrogen or estrogen-progesterone therapy.
(4). O (Outcome): the primary outcomes included clinical pregnancy rates and live birth rates; secondary outcomes included endometrial thickness, endometrial morphology, and serum estradiol (E2).
(5). S (Study Design): RCT.

#### Exclusion criteria

Studies on patients with severe mental disorders, animal experiments, non-Chinese or non-English publications, unavailable full text, and studies with incompatible data types were removed.

### 2.3. Literature screening and data extraction

Literature screening and data extraction were carried out independently by 2 researchers. Any discrepancies that arose during this phase were addressed through collaborative discussion between the researchers or, if necessary, by a 3rd researcher. In cases where the data in the articles were found to be insufficient or ambiguous, efforts were made to contact the corresponding authors to gather additional clarifying information through email or telephone communication. The collected studies were then organized using Endnote X9, with duplicate entries eliminated. Initially, the titles and abstracts were reviewed, and subsequently, the full texts for those studies that met the preliminary criteria were checked. The data extracted encompassed both baseline characteristics, such as the 1st author’s name, year of publication, country of origin, participant age, sample size, and details of interventions in both treatment and control groups, as well as outcome measures including clinical pregnancy rates, live birth rates, endometrial thickness, endometrial morphology, and E2 levels.

### 2.4. Quality assessment

The assessment of the risk of bias within the selected studies was conducted utilizing the updated Cochrane Risk of Bias tool (RoB-2). This tool facilitated the evaluation of various sources of bias, including those related to the randomization process, adherence to the planned interventions, completeness of outcome data, accuracy of outcome measurement, and the possibility of selective reporting. Studies were categorized into 3 categories: low risk of bias, some concerns, or high risk of bias. In instances where disagreements occurred, they were resolved through mutual agreement between the 2 reviewers or, if needed, by a third reviewer.

### 2.5. Data analysis

The meta-analysis was conducted utilizing Review Manager5.3 software (Version 5.3.5; The Cochrane Collaboration, Copenhagen, Denmark). For binary outcomes, the risk ratio (RR) along with its 95% confidence interval (CI) was employed. For continuous variables, the standardized mean difference (SMD) and its 95% CI were applied when there was a discrepancy in the units of measurement; otherwise, the mean difference (MD) and its 95% CI were utilized. The *I²* statistic served to assess statistical heterogeneity. An *I²* value of <50% suggested negligible heterogeneity, and thus a fixed-effects model was utilized. An *I²* value of 50% or greater along with a *P*-value <.05 denoted significant heterogeneity, and thereby a random-effects model was leveraged. Subgroup analyses were conducted to pinpoint the origins of heterogeneity. Sensitivity analysis was conducted using Stata 18.0 (StataCorp LLC, College Station) to explore the stability of the included studies, and publication bias was assessed through funnel plots and Egger test.

## 3. Results

### 3.1. Selection of studies

**Figure 1.**
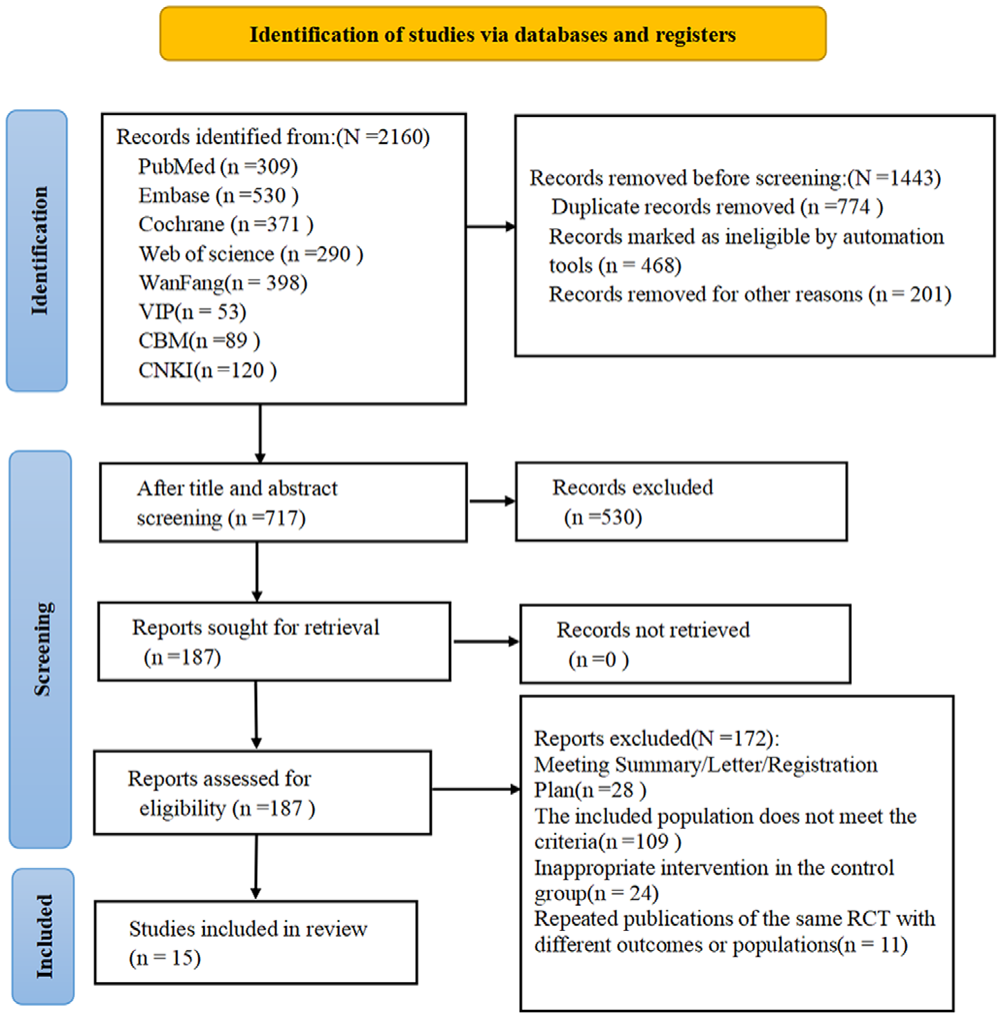
PRISMA flow diagram.

In line with the search strategy, a cumulative total of 2160 studies were identified. After excluding 774 duplicate studies, 468 records automatically marked as invalid were excluded, 201 studies were removed for other reasons, and 530 studies were excluded after reading titles and abstracts. Additionally, 172 studies were excluded due to being conference abstracts/letters/registration plans, having a population that did not meet the criteria, inappropriate interventions in the control group, or being duplicate publications of the same RCT with different results or populations. Finally, 15 studies were included (Fig. 1).

### 3.2. Study characteristics

The 15 eligible studies^[16–30]^ were all published in Chinese from 2018 to 2025. Despite the absence of limitations on research locations, each of these studies was carried out in China. The sample sizes varied between 15 and 61 participants across the different investigations. There were 5 studies on acupuncture + moxibustion treatment,^[16,17,19,20,30]^ 3 studies on regular acupuncture,^[24,26,27]^ 2 studies on electroacupuncture + warming acupuncture treatment,^[21,22]^ 1 study on electroacupuncture + milli-fire needle treatment,^[18]^ 1 study on needling + moxibustion treatment,^[23]^ 2 study on warming acupuncture treatment,^[25,29]^ and 1 study on electroacupuncture treatment.^[28]^ All studies reported outcome indicators, among which 12 studies reported endometrial thickness,^[16,18,19,21–25,27–30]^ 12 studies reported clinical pregnancy rates,^[16,18,21–30]^ 6 studies reported endometrial type,^[22,23,25,27–29]^ 3 studies reported live birth rates,^[22,26,28]^ and 4 studies reported E2 results.^[17,19,20,24]^ The features of the studies included are presented in Table 1.

**Table 1:** Basic information on included studies.

Study	Year	Country	Random method	Age (yr) (mean ± SD)		Cases		Intervention		Course of treatment (menstrual cycles)	Outcome
				T	C	T	C	T	C		
Wang	2021	China	(1)	33 ± 4	35 ± 5	15	15	(3) + C	(10)	2	(A)(C)
Xv	2023A	China	(1)	31.625 ± 4.288	30.875 ± 4.272	16	16	(3) + C	(11)	2	(E)
Qian	2022	China	(1)	35.05 ± 4.87	33.27 ± 3.70	30	30	(4) + C	(10)	3	(A)(C)
Shen	2023	China	(1)	28.43 ± 1.36	28.42 ± 1.35	31	31	(3) + C	(11)	2	(C)(E)
Xv	2023B	China	(1)	32.33 ± 0.84	31.25 ± 0.73	24	24	(3) + C	(11)	2	(E)
Xv	2018	China	(2)	33 ± 5	35 ± 5	36	36	(5) + C	(10)	3	(A)(C)
Xing	2023	China	(1)	34 ± 2	34 ± 2	36	36	(5) + C	(11)	3	(A)(B)(C)(D)
Xue	2021	China	(1)	35 ± 5	34 ± 4	37	37	(6) + C	(10)	3	(A)(C)(D)
Jin	2022	China	(1)	32.06 ± 4.25	31.59 ± 3.47	61	61	(7) + C	(11)	3	(A)(C)(E)
Ma	2018	China	(1)	30 ± 3	31 ± 4	30	30	(8) + C	(11)	1	(A)(B)(D)
You	2023	China	(1)	32.94 ± 1.79	33.29 ± 3.84	35	35	(7) + C	(11)	3	(A)(B)
Ma	2019	China	(1)	30.04 ± 2.98	30.5 ± 3.71	35	35	(7) + C	(11)	1	(A)(C)(D)
Deng	2024	China	(1)	35.52 ± 6.74	35.68 ± 7.25	51	49	(9) + C	(11)	1	(A)(B)(C)(D)
Fang	2022	China	(1)	32.55 ± 4.25	32.39 ± 4.47	29	28	(8) + C	(11)	2	(A)(C)(D)
Zhao	2025	China	(1)	30 ± 3	31 ± 4	50	50	(6) + C	(10)	2	(A)(C)

*Notes*: Random method: (1) random number table method; (2) random; intervention: (3) electroacupuncture + moxibustion; (4) electroacupuncture + milli-fire needle; (5) electroacupuncture + warming acupuncture; (6) acupuncture + moxibustion; (7) acupuncture; (8) warming acupuncture; (9) electroacupuncture; (10) estrogen; (11) estrogen–progesterone; outcome: (A): clinical pregnancy rate; (B): live birth rate; (C): endometrial thickness; (D): endometrial morphology; (E): E2.

C = control group, SD = standard deviation, T = treatment group.

### 3.3. Quality assessment of included studies

**Figure 2.**
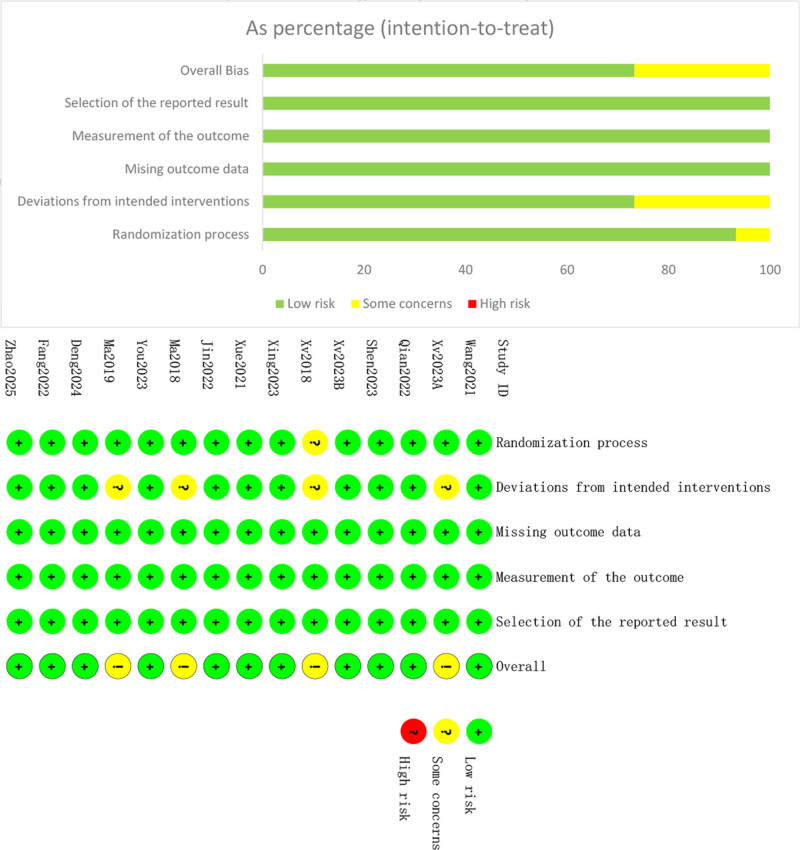
Risk of bias diagram.

Out of the 15 RCTs evaluated by the RoB-2 tool, 11 were classified as having a low risk of bias, whereas 4 exhibited a moderate risk of bias. In 1 study,^[21]^ the risk of randomization bias was described merely with the term “random,” lacking a clear explanation of the specific methods employed, which is considered to carry a certain risk of bias; in 4 studies,^[17,21,25,27]^ appropriate analyses were not employed to assess the impact of intervention allocation, also indicating a certain risk of bias. Details of the bias risk assessment are depicted in Figure 2.

### 3.4. Meta-analysis results

#### 3.4.1. Clinical pregnancy rate

**Figure 3.**
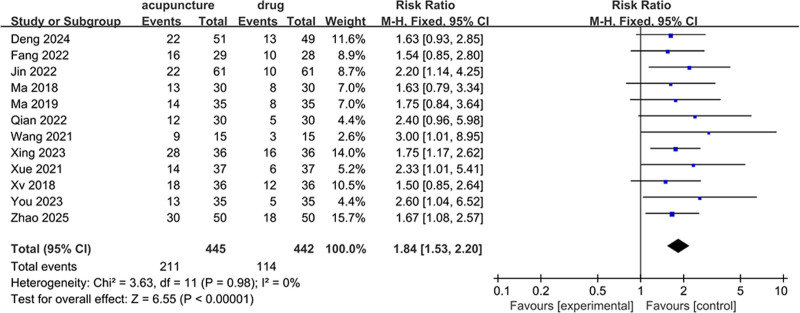
Forest plot of clinical pregnancy rates.

Twelve studies, comprising a total of 887 patients, with 445 in the acupuncture and moxibustion treatment group and 442 in the control group, reported on clinical pregnancy rates. These studies demonstrated no significant heterogeneity (*I*² = 0.0%, *P* = .98), and thus a fixed-effects model was adopted for analysis. The findings suggested that acupuncture and moxibustion treatment was associated with a higher rate of clinical pregnancies among patients with RIF compared to the control group (RR = 1.84, 95% CI = 1.53–2.20, *P* < .05) (Fig. 3).

#### 3.4.2. Live birth rate

**Figure 4.**
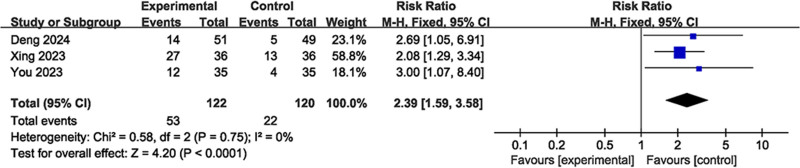
Forest plot of live birth rate.

Three studies, which included a total of 242 patients, with 122 assigned to the acupuncture and moxibustion treatment group and 120 to the control group, reported on live birth rates. These studies indicated no significant heterogeneity (*I*² = 0.0%, *P *= .75), and a fixed-effects model was utilized. The results indicated that the effects of the intervention on the live birth rate for patients experiencing RIF were more positive in the acupuncture and moxibustion treatment group compared to the control group. (RR = 2.39, 95% CI = 1.59–3.58, *P* < .05) (Fig. 4).

#### 3.4.3. Endometrial thickness

**Figure 5.**
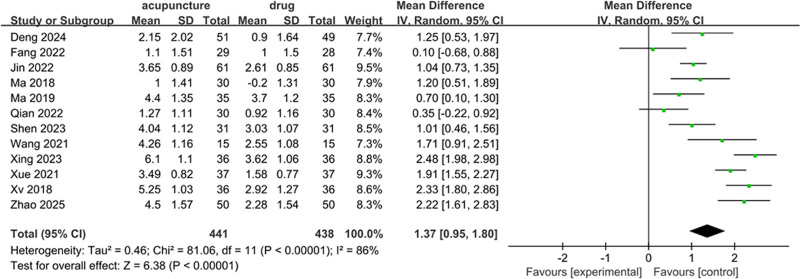
Forest plot of endometrial thickness.

Twelve studies, encompassing 879 participants, reported data on endometrial thickness, with 441 allocated to the acupuncture and moxibustion treatment group and 438 to the control group. Given the considerable heterogeneity observed across the studies (*I*² = 86%, *P* < .00001), a random-effects model was utilized for the analysis. The findings indicated that acupuncture and moxibustion treatment was more effective in enhancing endometrial thickness in patients with RIF than the control intervention (MD = 1.37, 95% CI = 0.95–1.80, *P* < .05) (Fig. 5).

##### 3.4.3.1. Subgroup analysis

To delve deeper into the observed heterogeneity, a subgroup analysis was conducted. This analysis revealed that acupuncture and moxibustion treatment was efficacious across different durations. Among these treatments, the intervention over 3 menstrual cycles is more effective in improving the endometrial thickness of patients experiencing RIF (MD = 1.62, 95% CI: 0.92–2.33, *P* < .001, *I*^2^ = 92%). In comparison to both continuous estrogen use (MD = 1.71, 95% CI: 1.03–2.38, *P* < .001, *I*^2^ = 87%) and sequential therapy with estrogen and progesterone (MD = 0.93, 95% CI: 0.67–1.20, *P* < .001, *I*^2^ = 26%), acupuncture and moxibustion treatment could effectively increase endometrial thickness. Regarding the selection of acupuncture types, electroacupuncture + moxibustion, electroacupuncture + warming acupuncture, acupuncture + moxibustion, acupuncture, and electroacupuncture all effectively increased the endometrial thickness in patients with RIF, with electroacupuncture + warming acupuncture showing particularly significant effects (MD = 2.41, 95% CI: 2.05–2.77, *P* < .001, *I*^2^ = 0%). In terms of acupoint selection methods, selecting acupoints based on the root and branch of meridian theory, staged acupuncture method, and ordinary point selection method could all effectively increase the endometrial thickness in patients with RIF, with the staged acupuncture method showing particularly significant effects (MD = 2.41, 95% CI: 2.05–2.77, *P* < .001, *I*^2^ = 0%). Based on the subgroup analysis heterogeneity test results, we were unable to identify the source of heterogeneity affecting the outcome indicator of endometrial thickness (Table 2).

**Table 2:** Subgroup analysis of the influence of acupuncture and moxibustion on endometrial thickness in patients with RIF.

Intervention characteristics	Study quantity	Total number of cases	Heterogeneity		MD (95% CI)	Meta-analysis results		Meta-regression	
			*I*^2^ (%)	*P*-value		*Z*	*P*	*I*^2^ (%)	*P*-value
Treatment cycles									
1 menstrual cycle	Ma (2018), Ma (2019), Deng (2024)	230	0	.42	1.01 (0.63 to 1.39)	5.17	<.00001	13.8	.31
2 menstrual cycles	Wang (2021), Shen (2023), Fang (2022), Zhao (2025)	249	85	.0002	1.27 (0.41 to 2.14)	2.88	.004		
3 menstrual cycles	Qian (2022), Xv (2018), Xing (2023), Xue (2021), Jin (2022)	400	92	<.00001	1.62 (0.92 to 2.33)	4.52	<.00001		
Basic treatment									
Continuous oral estrogen	Wang (2021), Qian (2022), Xv (2018), Xue (2021), Zhao (2025)	336	87	<.00001	1.71 (1.03 to 2.38)	4.95	<.00001	77.1	.04
Sequential therapy with estrogen and progesterone	Shen (2023), Xing (2023), Jin (2022), Ma (2018), Ma (2019), Deng (2024), Fang (2022)	507	26	.24	0.93 (0.67 to 1.20)	6.89	<.00001		
Type of acupuncture types									
Electroacupuncture + moxibustion	Wang (2021), Shen (2023)	92	50	.16	1.30 (0.62 to 1.97)	3.77	.0002	91.0	<.00001
Electroacupuncture + milli-fire needle	Qian (2022)	60	–	–	0.35 (-0.22 to 0.92)	1.19	.23		
Electroacupuncture + warming acupuncture	Xv (2018), Xing (2023)	144	0	.69	2.41 (2.05 to 2.77)	12.95	<.00001		
Acupuncture + moxibustion	Xue (2021), Zhao (2025)	174	0	.39	1.99 (1.68 to 2.30)	12.53	<.00001		
Acupuncture	Jin (2022), Ma (2019)	182	0	.32	0.97 (0.69 to 1.24)	6.92	<.00001		
Warming acupuncture	Ma (2018), Fang (2022)	117	77	.04	0.67 (-0.41 to 1.74)	1.21	.23		
Electroacupuncture	Deng (2024)	100	–	–	1.25 (0.53 to 1.97)	3.40	.0007		
Acupoint selection									
Selecting acupoints based on the root and branch of meridian theory	Wang (2021), Shen (2023), Deng (2024)	192	0	.37	1.24 (0.85 to 1.62)	6.34	<.00001	90.5	<.00001
Tongyuan acupuncture technique	Qian (2022)	60	–	–	0.35 (-0.22 to 0.92)	1.19	.23		
Staged acupuncture	Xv (2018), Xing (2023)	144	0	.69	2.41 (2.05 to 2.77)	12.95	<.00001		
Tongyuan acupuncture technique + staged acupuncture	Xue (2021), Jin (2022)	196	92	.0003	1.47 (0.62 to 2.32)	3.38	.0007		
Ordinary acupoint selection	Ma (2018), Ma (2019), Fang (2022), Zhao (2025)	287	86	.0001	1.07 (0.19 to 1.95)	2.39	.02		

RIF = recurrent implantation failure.

#### 3.4.4. Endometrial morphology

**Figure 6.**
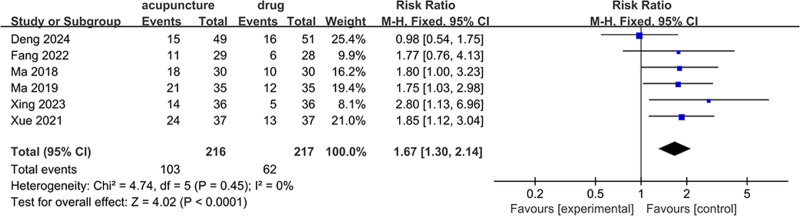
Forest plot of endometrial morphology.

Six studies utilized type A endometrium as a criterion for evaluation, encompassing a total of 433 patients, with 216 in the acupuncture and moxibustion group and 217 in the control group. These studies displayed a modest level of heterogeneity (*I*^2^ = 0%, *P* = .45), and thus a fixed-effects model was leveraged for the analysis. The results revealed that the acupuncture and moxibustion treatment group had a notably higher rate of type A endometrium compared to the control group (RR = 1.67, 95% CI = 1.30–2.14, *P* < .05) (Fig. 6).

#### 3.4.5. E2 levels

**Figure 7.**
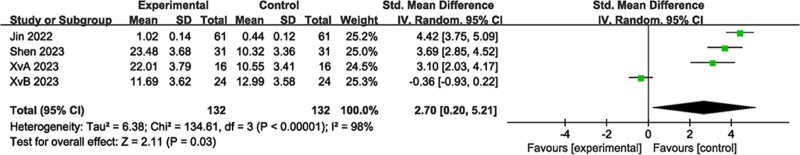
Forest plot of E2. E2 = serum estradiol.

Four studies, comprising 264 patients, with 132 in the acupuncture and moxibustion treatment group and 132 in the control group, documented serum E2 levels. Given the pronounced heterogeneity among the studies (*I*² = 98%, *P* < .00001), a random-effects model was deemed appropriate for analysis. The findings suggested that acupuncture and moxibustion treatment had a more favorable impact on E2 levels in patients with RIF than the control intervention (SMD = 2.70, 95% CI = 0.20–5.21, *P* < .05) (Fig. 7).

##### 3.4.5.1. Subgroup analysis

To explore the sources of heterogeneity for this outcome indicator, we conducted subgroup analyses based on the treatment cycle, acupuncture types, and acupoint selection methods. The results revealed that, in contrast to the control group, the acupuncture treatment of 3 menstrual cycles (SMD = 4.42, 95% CI: 3.75–5.09), the needling technique (SMD = 4.42, 95% CI: 3.75–5.09), and the Tongyuan acupuncture technique combined with a staged acupuncture approach (SMD = 4.42, 95% CI: 3.75–5.09) all enhanced E2 levels in patients with RIF. However, we did not find the source of heterogeneity for the E2 level outcome (Table S1, Supplemental Digital Content, https://links.lww.com/MD/Q910).

### 3.5. Sensitivity analysis

A sensitivity analysis was performed on all outcome indicators, revealing that the results were overall stable and reliable (Figures S1–S5, Supplemental Digital Content, https://links.lww.com/MD/Q910).

### 3.6. Publication bias

For outcome indicators reported in more than 10 included studies,^[31]^ namely clinical pregnancy rate and endometrial thickness, publication bias was assessed. The results showed that the funnel plots of both outcomes were roughly symmetrical (Figures S6 and S7, Supplemental Digital Content, https://links.lww.com/MD/Q910). Further Egger test showed that the *P*-value for clinical pregnancy rate was .010 (*P* < .05), indicating the presence of publication bias. Therefore, a trim-and-fill method was used for correction. The *P* values before and after trimming were both < .001 (Figure S8, Supplemental Digital Content, https://links.lww.com/MD/Q910), with no significant changes in the results, indicating that although there was publication bias, it did not affect the reliability of the results. For the outcome of endometrial thickness, Egger test showed *P* = .721 (*P* > .05), indicating no publication bias.

## 4. Discussion

This systematic review has assessed the impact of acupuncture and moxibustion treatment on individuals with RIF. Meta-analysis indicates that acupuncture and moxibustion therapy is more effective than control interventions in enhancing clinical pregnancy rates and live birth rates in patients experiencing RIF, increasing endometrial thickness, improving endometrial morphology, and elevating E2 levels. The subgroup analysis showed that for improving endometrial thickness in RIF patients, acupuncture with a treatment period of 3 menstrual cycles, the use of electroacupuncture and warming acupuncture, and the acupuncture point selection method of the staged acupuncture were more effective. For increasing E2 levels in RIF patients, acupuncture with a treatment period of 3 menstrual cycles, the type selection of needling, and the acupoint selection method of Tongyuan acupuncture technique combined with staged acupuncture were all effective interventions.

Acupuncture and moxibustion therapy can increase clinical pregnancy rates and live birth rates in patients experiencing RIF. This is consistent with the results reported in multiple studies.^[32–36]^ Acupuncture and moxibustion can improve uterine microcirculation by regulating the sympathetic nervous system, thereby optimizing the maternal internal environment, promoting the structural repair of the endometrium, and adjusting endometrial morphology.^[37]^ This approach enhances endometrial receptivity, providing favorable conditions for embryo implantation and potentially improving pregnancy outcomes to a certain extent.^[11]^ In addition, acupuncture and moxibustion therapy helps to increase the release of β-endorphins, endorphins, enkephalins, serotonin, and other neurochemicals through the regulation of peripheral nervous systems, alleviating mental stress and reducing patients’ feelings of anxiety and depression, thereby improving pregnancy outcomes.^[38,39]^

Acupuncture and moxibustion therapy enhances endometrial thickness, improves endometrial morphology, and elevates E2 levels in patients dealing with RIF. Research suggests that endometrial thickness serves as an important parameter for assessing endometrial receptivity.^[40]^ Multiple studies indicate that a reduction in the clinical pregnancy rate occurs when the endometrial thickness measures <8 millimeter during fresh embryo transfer or <7 millimeter during frozen-thawed embryo transfer.^[41]^ The observed increase in endometrial thickness following acupuncture and moxibustion treatment could be due to its capacity to upregulate the expression of a range of adhesion molecules and cytokines, such as integrin αvβ3, Homeobox A10 gene, heparin-binding epidermal growth factor, estrogen receptor α, and progesterone receptor. This upregulation, via multiple molecular targets, facilitates the formation of pinopodes in the endometrium, which leads to a dense accumulation of the endometrial basilar layer and an increased number of blood vessels and glands, ultimately enhancing the thickness of the endometrium.^[11,42,43]^ In terms of improving endometrial morphology, a review of acupuncture and moxibustion therapy aimed at enhancing endometrial receptivity over the past decade mentioned that this treatment can lead to improvements in endometrial morphology,^[44]^ aligning with our findings. However, the report by Li is inconsistent with ours,^[12]^ which may be related to the greater number of articles and patients included in our study. Regarding the outcome indicator of E2 levels, research indicates that under conditions characterized by low sex hormone levels, acupuncture and moxibustion therapy is capable of stimulating the activity of aromatase, enhancing the synthesis of neuropeptide Y, boosting blood circulation within the ovaries, and thereby elevating estrogen levels. Appropriate levels of estrogen and progesterone contribute positively to pregnancy.^[45]^ A study by Wu reported that acupuncture and moxibustion therapy can increase E2 levels in patients dealing with infertility.^[46]^ The findings from Cao’s study also reported that acupuncture and moxibustion therapy was superior to the non-acupuncture and moxibustion group in increasing E2 levels.^[47]^

The subgroup analysis of acupuncture and moxibustion treatment for RIF patients showed that an intervention period of 1 to 3 menstrual cycles can increase the endometrial thickness in RIF patients, with better results observed for a treatment duration of 3 menstrual cycles. In the systematic review by Zhu et al, the acupuncture treatment periods in their included studies range from 1 to 3 menstrual cycles, and the effectiveness of the treatment is also reported,^[32]^ which is consistent with our results. A study by Li et al indicated that a higher dosage of acupuncture and moxibustion treatment seemed to improve pregnancy outcomes in patients undergoing IVF–ET.^[48]^ A study by Wang et al emphasized the importance of the acupuncture treatment course, suggesting that the improvement in pregnancy outcomes is due to the cumulative effect of acupuncture treatment, akin to dose dependence.^[49]^ Several studies indicate that patients with RIF frequently exhibit kidney qi deficiency and qi-blood deficiency, both of which are categorized as deficiency syndromes. In clinical practice, when addressing deficiency syndromes through acupuncture, it is advisable to use a small amount of stimulation, maintain a prolonged retention time, and extend the duration of the treatment course.^[50]^ However, some studies indicate that the length of treatment is not necessarily positively correlated with the outcomes following acupuncture. When the stimulation time surpasses a specific threshold, effective stimulation may become ineffective, or even detrimental, resulting in adverse effects.^[50]^ In terms of acupuncture and moxibustion types, electroacupuncture + moxibustion, electroacupuncture + warming acupuncture, acupuncture + moxibustion, acupuncture, and electroacupuncture can effectively increase endometrial thickness in RIF patients, with electroacupuncture + warming acupuncture showing particularly significant effects. A meta-analysis conducted by Zhang et al regarding the treatment effects of various acupuncture methods for chronic nonspecific low back pain recommended the use of warming acupuncture and electroacupuncture.^[51]^ Li et al demonstrated that the combination of electroacupuncture and warming acupuncture provided a relatively effective therapy for traumatic nerve injury.^[52]^ Nevertheless, the current body of research is sparse when it comes to understanding how various acupuncture modalities affect the treatment outcomes for RIF. It is recommended that future studies delve into this aspect to expand the knowledge base in this field. We believe that the effectiveness of combining electroacupuncture and warming acupuncture is due to their simultaneous integration of the benefits of electroacupuncture, hand acupuncture, and moxibustion. This combination directly or indirectly stimulates the sensory nerve fibers associated with the acupoint through electrical, mechanical, and thermal stimulation, resulting in various effects.^[53]^ Choosing staged acupuncture as the acupoint selection method is the best therapeutic strategy, though research in this area is relatively limited. Through the lens of traditional Chinese medicine, which is grounded in the principles of yin-yang and the 5-element theory, a treatment strategy that is phased and responsive to these cyclical fluctuations is necessary given the menstrual cycle’s ebb and flow in women’s vital energy, blood, yin, and yang. This approach should take into account both physiological characteristics and the different needs of the implantation cycle during assisted reproductive interventions, aiming to achieve balance between yin and yang, and harmony in qi and blood, and improve pregnancy outcomes.^[54]^

### 4.1. Limitations

This study presents several limitations. Firstly, while we do not impose restrictions on the research locations of the included articles, all the included articles originate from China. Further high-quality research is required to confirm the treatment effects across various ethnic groups. Secondly, we excluded non-Chinese and non-English literature, which may introduce language bias and overlook significant contributions written in other languages. Thirdly, all the included studies were written in Chinese, which may not reach an adequate level of quality. Fourthly, although we conducted subgroup analyses for endometrial thickness, the number of included studies in each category is limited. For example, in the subgroup analysis of acupuncture types and acupuncture point selection methods, the sample size in each subgroup is small, resulting in low evidence quality and limited generalizability of the obtained results. Consequently, our results can only provide a reference for clinical practice, and caution is advised when interpreting the outcomes. Fifth, the outcome indicator of endometrial thickness and E2 levels exhibited high levels of heterogeneity. Although subgroup analyses were performed based on treatment cycles, baseline therapies, acupuncture types, and acupoint selection methods, the primary source of heterogeneity remains unclear. Consequently, future high-quality studies containing complete baseline data are required to further explore potential influencing factors.

## 5. Conclusion

Acupuncture and moxibustion therapy have been shown to be effective in enhancing clinical pregnancy rates, live birth rates, endometrial thickness, improving endometrial morphology, and increasing E2 levels in patients with RIF. This finding provides a theoretical basis for acupuncture treatment in patients with RIF. Nevertheless, given the constraints within the current body of studies, additional large-scale, multi-centric, and rigorous clinical trials are warranted to investigate the variability in acupuncture’s therapeutic effects across different age groups and to delve deeper into the determinants that affect treatment outcomes.

## Author contributions

**Conceptualization:** Xuzhen Cheng.

**Formal analysis:** Junjun Chen, Yan Lyu.

**Investigation:** Junjun Chen, Yan Lyu.

**Methodology:** Junjun Chen.

**Resources:** Xuzhen Cheng.

**Supervision:** Fang Zhang.

**Writing – original draft:** Junjun Chen.

**Writing – review & editing:** Yan Lyu.

## Supplementary Material


