# Supplementary material for: The impact of acupuncture and moxibustion treatment in individuals with recurrent implantation failures: A systematic review and meta-analysis
Source: Medicine (Baltimore). 2025 Dec 19;104(51):e46587. doi: 10.1097/MD.0000000000046587 (PMC12727353; doi:10.1097/MD.0000000000046587)
Supplement: Supplementary file 1 [file medi-104-e46587-s001.pdf]

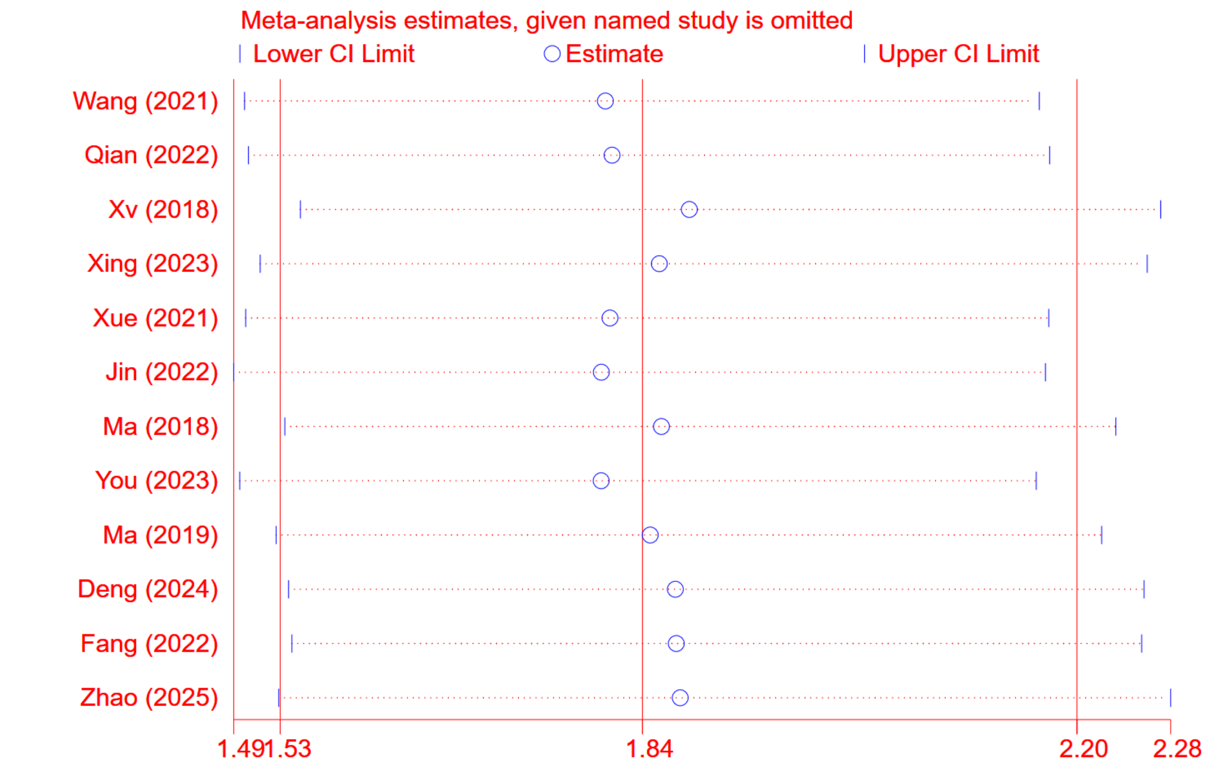

Figure S1 Sensitivity analysis of clinical pregnancy rate

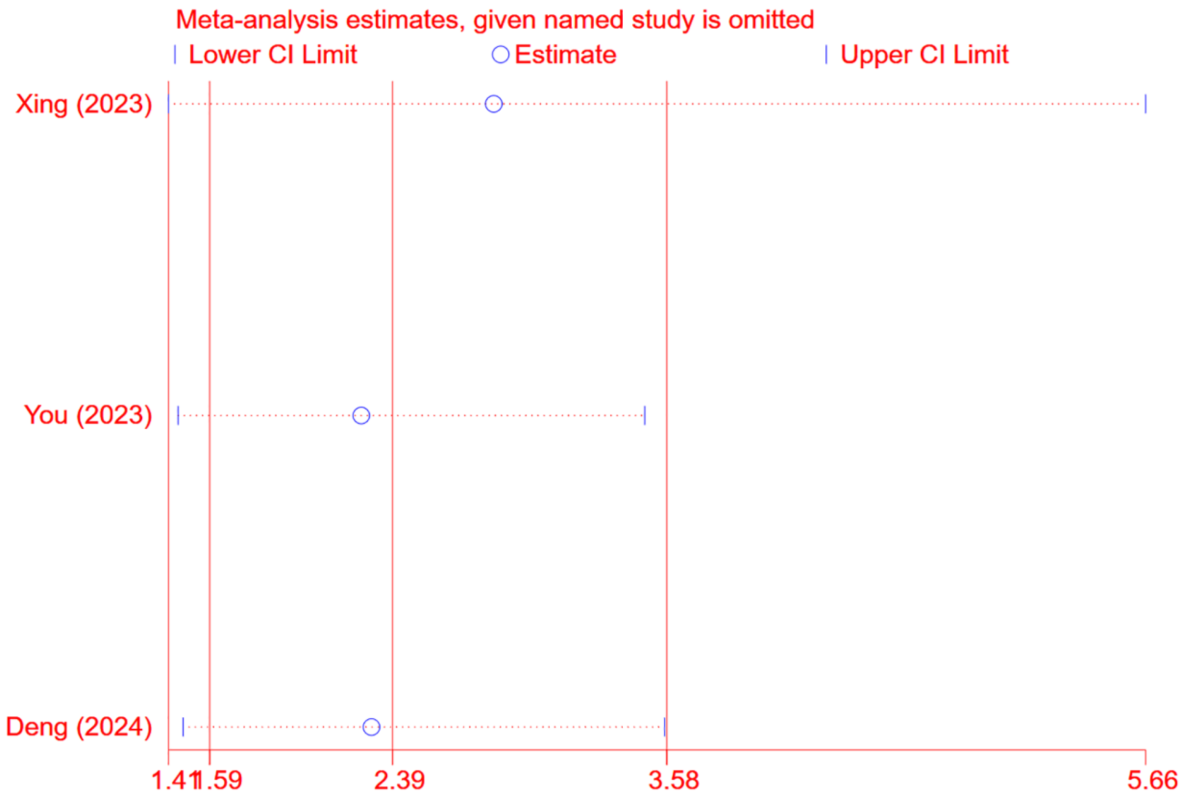

Figure S2 Sensitivity analysis of live birth rate

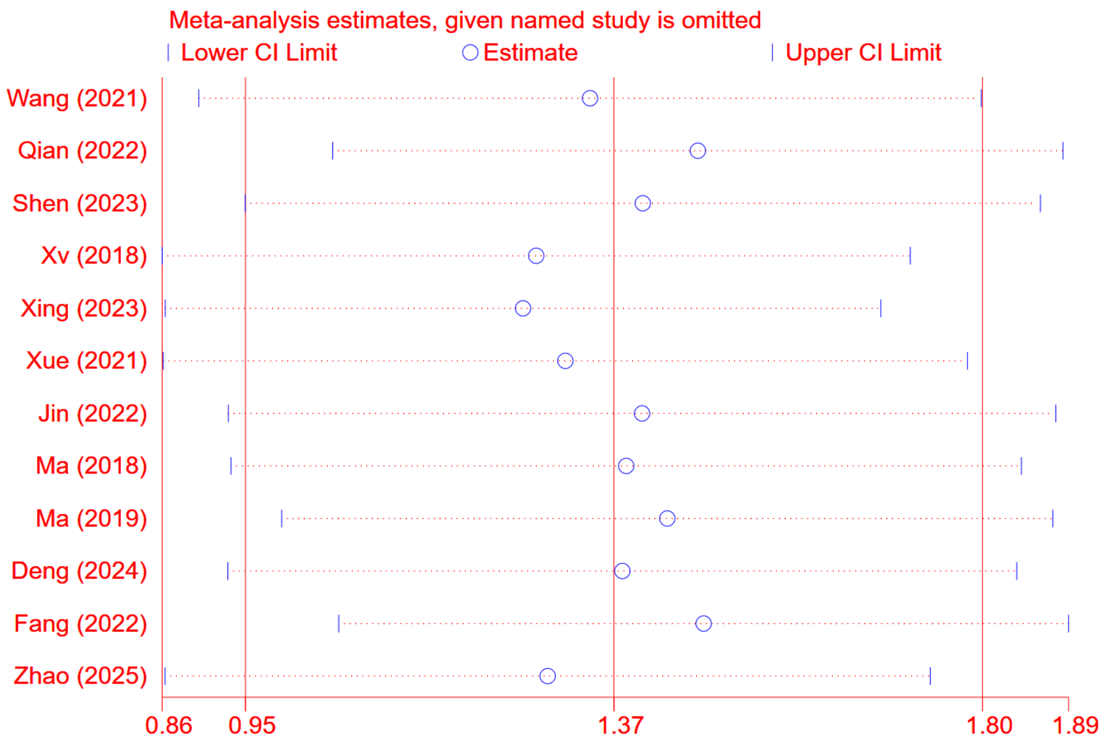

Figure S3 Sensitivity analysis of endometrial thickness

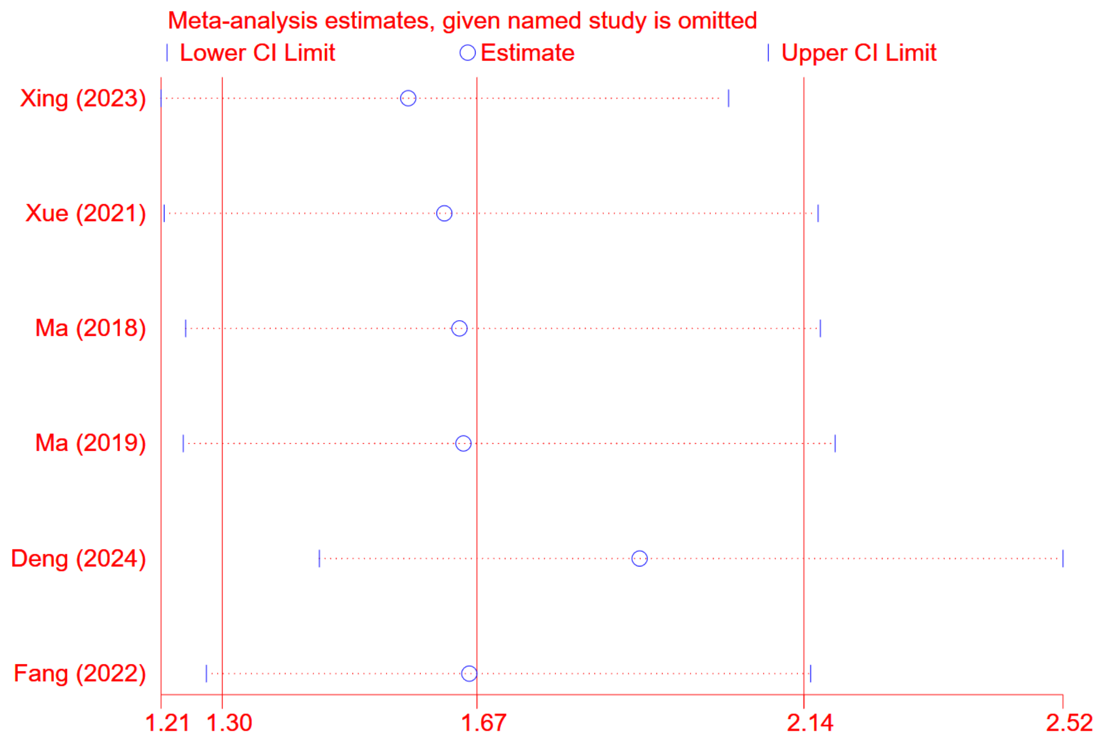

Figure S4 Sensitivity analysis of endometrial morphology

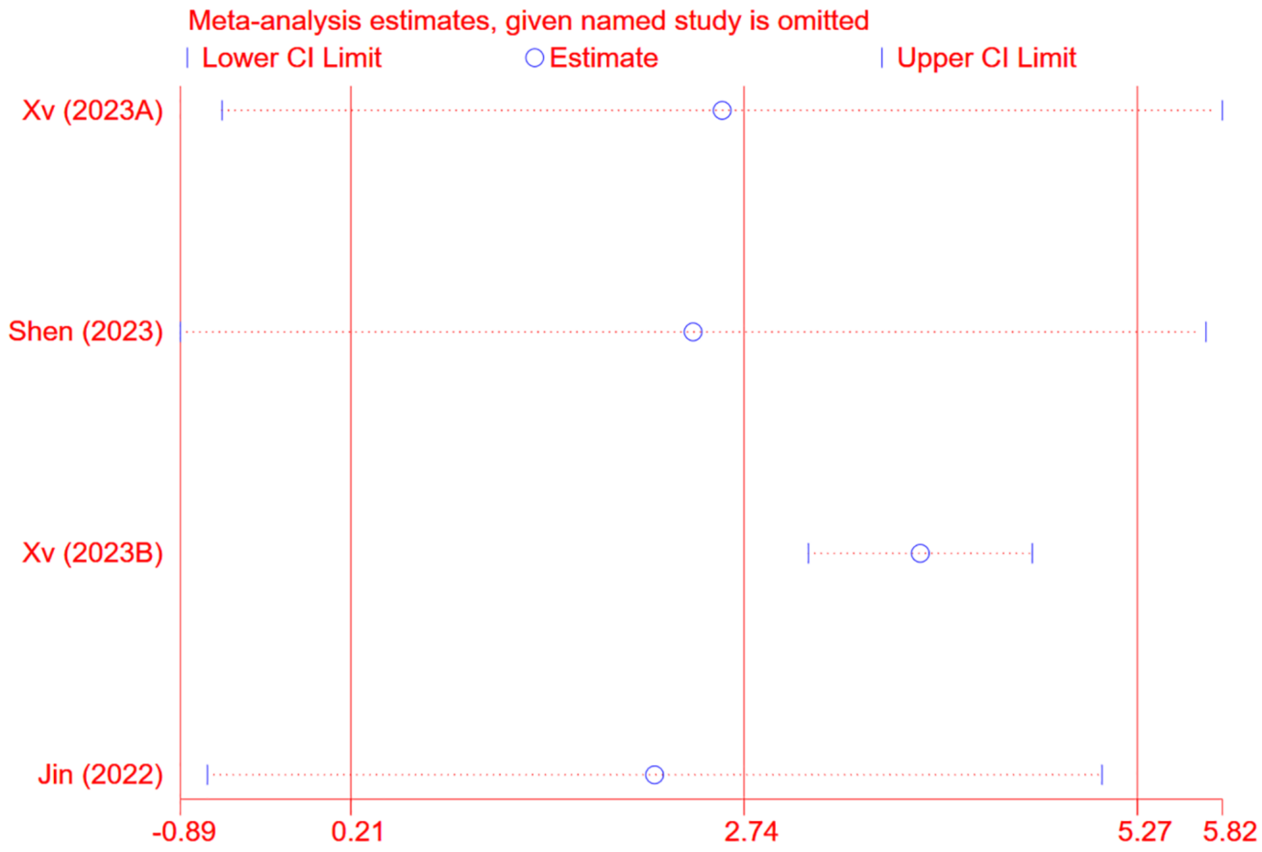

Figure S5 Sensitivity analysis of E2 levels

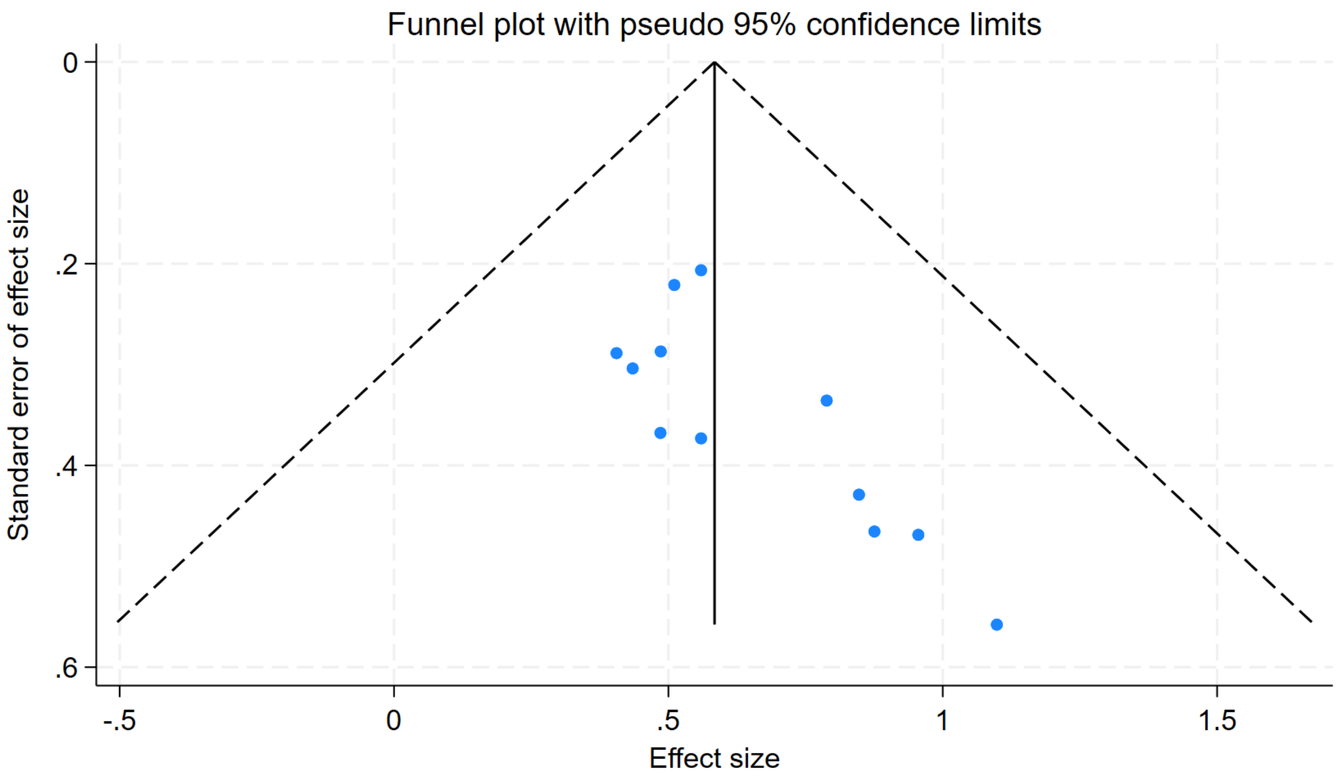

Figure S6 Funnel plot of clinical pregnancy rate

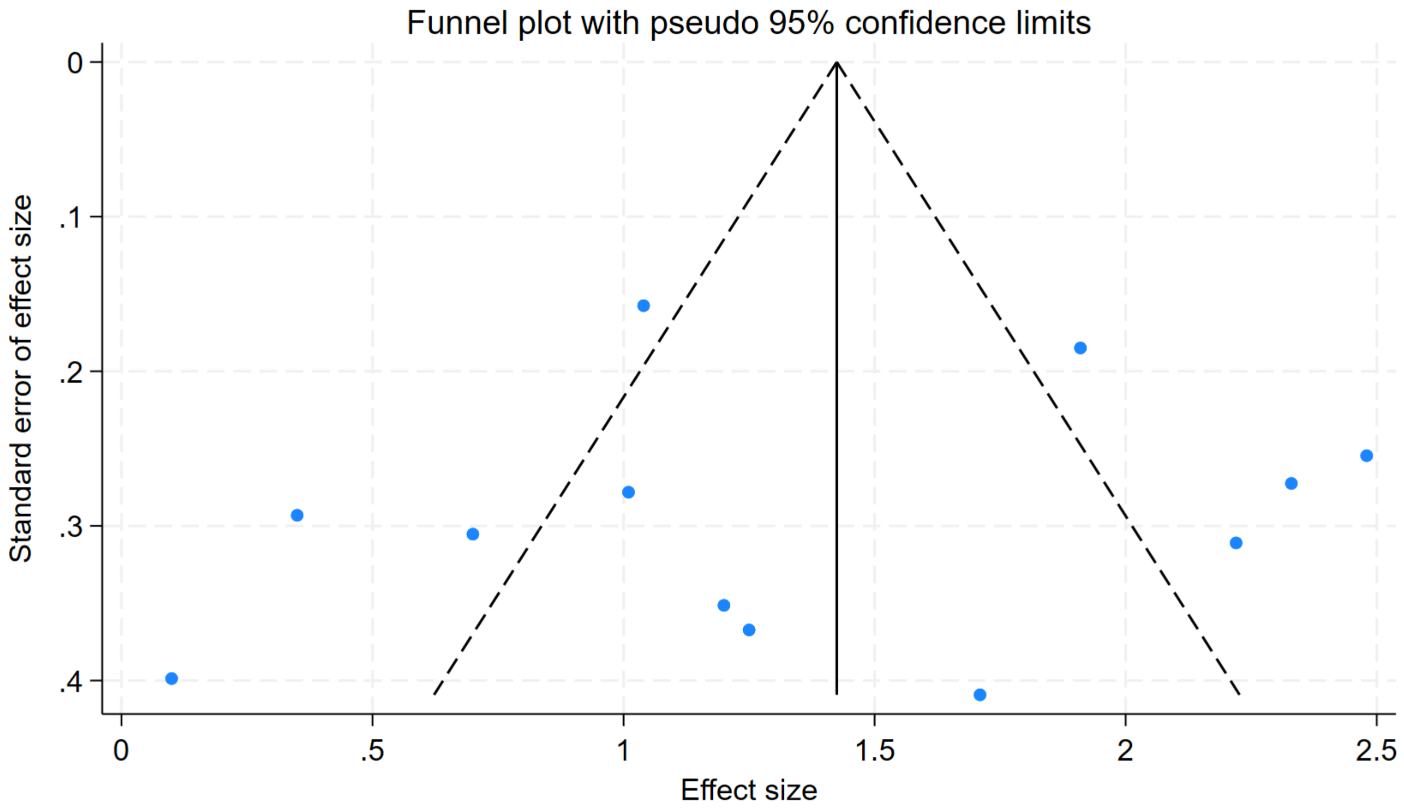

Figure S7 Funnel plot of endometrial thickness

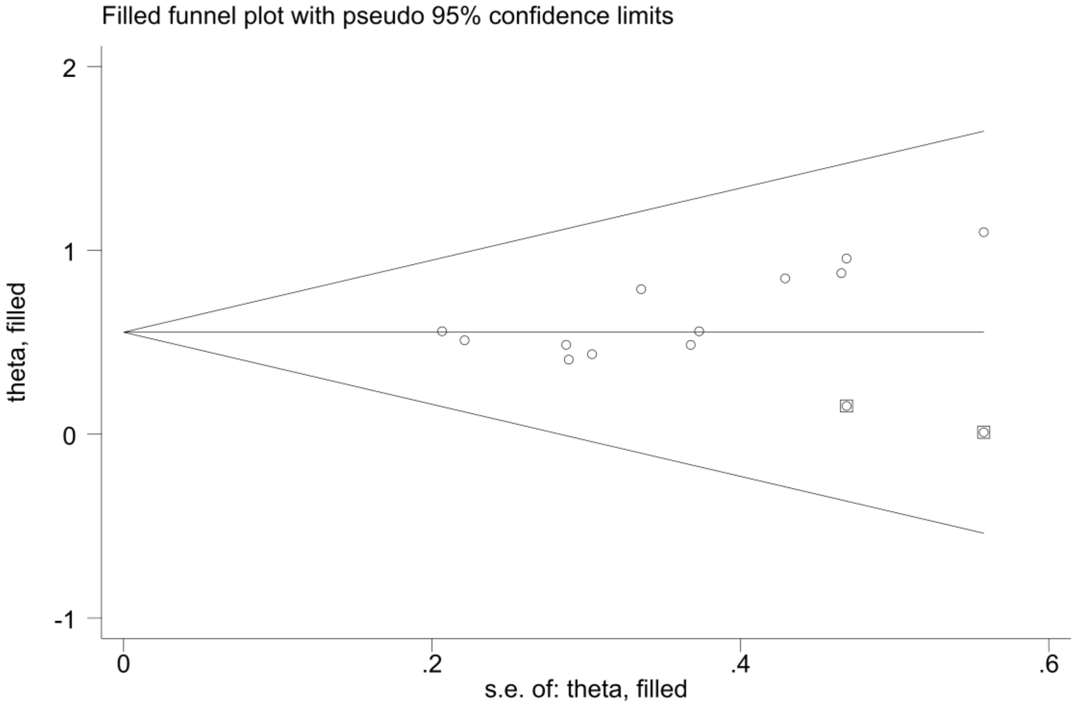

Figure S8 Publication bias assessment for clinical pregnancy rate

Table S1 Subgroup analysis of E2

**Intervention characteristics**	**Study quantity**	**Total number of cases**	**Heterogeneity**		**MD(95%CI)**	**Meta-analysis results**		**Meta-regression**	
			I^2**(%)**	***P*-value**		**Z**	***P***	I^2**(%)**	***P*-value**
Treatment cycles									
2 menstrual cycles	Xv (2023A), Shen (2023), Xv (2023B)	142	97	<0.00001	2.13(-0.71,4.96)	1.47	0.14	58.1	0.12
3 menstrual cycles	Jin(2022)	122	-	-	4.42(3.75,5.09)	13.00	<0.00001		
Type of acupuncture									
Acupuncture+ moxibustion	Xv (2023A), Shen (2023), Xv (2023B)	142	97	<0.00001	2.13(-0.71,4.96)	1.47	0.14	58.1	0.12
Acupuncture	Jin(2022)	122	-	-	4.42(3.75,5.09)	13.00	<0.00001		
Acupoint selection									
Selecting acupoints based on the root and branch of meridian theory	Xv (2023A), Shen (2023), Xv (2023B)	142	97	<0.00001	2.13(-0.71,4.96)	1.47	0.14	58.1	0.12
Tongyuan acupuncture technique +Staged acupuncture	Jin(2022)	122	-	-	4.42(3.75,5.09)	13.00	<0.00001		
